# Prevalence of child malnutrition at a university hospital using the World Health Organization criteria and bioelectrical impedance data

**DOI:** 10.1590/1414-431X20155012

**Published:** 2016-02-02

**Authors:** V.N. Pileggi, J.P. Monteiro, A.V.B. Margutti, J.S. Camelo

**Affiliations:** 1 Departamento de Puericultura e Pediatria, Faculdade de Medicina de Ribeirão Preto, Universidade de São Paulo, Ribeirão Preto, SP, Brasil; 2 Curso de Nutrição e Metabolismo, Faculdade de Medicina de Ribeirão Preto, Universidade de São Paulo, Ribeirão Preto, SP, Brasil; 3 Curso de Nutrição, Universidade de Ribeirão Preto, Ribeirão Preto, SP, Brasil

**Keywords:** Child malnutrition, Prevalence, Bioelectrical impedance

## Abstract

Malnutrition constitutes a major public health concern worldwide and serves as an indicator of hospitalized patients’ prognosis. Although various methods with which to conduct nutritional assessments exist, large hospitals seldom employ them to diagnose malnutrition. The aim of this study was to understand the prevalence of child malnutrition at the University Hospital of the Ribeirão Preto Medical School, University of São, Brazil. A cross-sectional descriptive study was conducted to compare the nutritional status of 292 hospitalized children with that of a healthy control group (n=234). Information regarding patients’ weight, height, and bioelectrical impedance (i.e., bioelectrical impedance vector analysis) was obtained, and the phase angle was calculated. Using the World Health Organization (WHO) criteria, 35.27% of the patients presented with malnutrition; specifically, 16.10% had undernutrition and 19.17% were overweight. Classification according to the bioelectrical impedance results of nutritional status was more sensitive than the WHO criteria: of the 55.45% of patients with malnutrition, 51.25% exhibited undernutrition and 4.20% were overweight. After applying the WHO criteria in the unpaired control group (n=234), we observed that 100.00% of the subjects were eutrophic; however, 23.34% of the controls were malnourished according to impedance analysis. The phase angle was significantly lower in the hospitalized group than in the control group (P<0.05). Therefore, this study suggests that a protocol to obtain patients’ weight and height must be followed, and bioimpedance data must be examined upon hospital admission of all children.

## Introduction

Malnutrition can be defined as an imbalance between the need and intake of essential nutrients. Both undernutrition and excess weight can impair growth and cognitive development, increase the risk of infections, and prolong wound healing. Moreover, malnutrition might have financial consequences for both the individual and health system because of a lengthening of the hospital stay (1). Therefore, this matter must be resolved worldwide.

The prevalence of malnutrition in hospitalized children varies largely among countries. According to a Brazilian study, 16.3% of children aged <5 years at admission exhibited undernutrition (2). In Australia, O’Connor et al. (3) observed a malnutrition rate of 5-27%. Joosten et al. (4) reported malnutrition in 6-14% of children. In a review in England, 11-45% of the children were found to have malnutrition (5). These differences might be due to the lack of standardization in risk assessment methodologies and screening of hospitalized children (6).

Anthropometric parameters enable the hospital staff to detect patients with malnutrition at admission. However, it is important that basic and/or disease diagnosis procedures and treatment protocols are used to recognize patients at high risk of developing malnutrition despite having an adequate nutritional status at admission.

Traditional bioelectric impedance analysis (BIA) involves predictive equations and new approaches, such as bioelectrical impedance vector analysis (BIVA) and phase angle (PA) (7) studies, which can be useful tools for evaluating a patient’s secondary nutritional status. Both BIVA and PA involve graphical analyses. BIVA comprises four different parameters, namely eutrophic, lean, cachectic, and obese and athletic (definitions of these parameters, provided by Piccoli et al. (7), do not follow the classic meanings), that do not require data regarding weight. The PA, which evaluates how membrane integrity is associated with prognosis, requires only the resistance (R) and reactance (Xc) values (8 9 10). Nagano et al. (11) suggested that the PA is a useful parameter for the nutritional assessment of body cell mass in stable pediatric patients; however, PA studies in children are scarce (8,11).

The Hospital das Clínicas, Faculdade de Medicina de Ribeirão Preto, Universidade de São Paulo (HCFMRP/USP) is a referral hospital in Brazil that mainly treats patients with severe and chronic diseases. However, no investigations to date have addressed the nutritional status of patients admitted to the pediatric wards. Malnutrition is a major public health concern; therefore, reducing the number of individuals with this condition is essential to improve patients’ prognosis and minimize hospitalization costs. The primary aim of this study was to determine the prevalence of malnutrition in the pediatric wards of HCFMRP/USP. In the secondary analysis, we evaluated whether the nursing staff performed anthropometric assessments in the wards by checking these data in the hospital system. A third objective of the present work was to assess patients’ nutritional status by BIVA and PA. We hypothesized that the prevalence of malnutrition at our tertiary hospital would be approximately 30% based on previous literature (3 4 5).

## Material and Methods

This descriptive study (cross-sectional cohort) included children and adolescents aged 1 month to 18 years. All patients were evaluated within the first 48 h of admission to the pediatric wards of HCFMRP/USP between February 2012 and February 2013 by a single researcher to avoid bias (VN Pileggi). The numbers of beds in the pediatric wards were as follows: 10 beds for gastroenterology, 9 for oncology, 7 for nephrology, 4 for pneumology, 4 for cardiology, 2 for endocrinology, 1 for rheumatology, and 1 for immunology. Concurrently, a control group comprising healthy children and adolescents who were followed up in a childcare ambulatory unit near the hospital was used for comparison purposes. The control group (aged 1 month and 18 years) was initially matched for age, sex, and socioeconomic status. The number of hospitalizations in the previous 3 years and the estimated prevalence of malnutrition of 30% helped us determine the sample size (n=292 for the hospitalized group).

The exclusion criteria were as follows: newborns, pediatric patients admitted to the pediatric intensive care unit, data collection after 48 h, patients with edema (except biochemically proven nutritional edema), patients with polytrauma, children in the emergency room, and children whose parents refused their participation in the study.

The anthropometric data collection assisted us with the nutritional status assessment. The patients’ history was obtained and recorded using a questionnaire (e.g., duration of breastfeeding and maternal school age). The cutoff points for the body mass index according to age (BMI/A) were determined according to the z-score of the WHO table of parameters (+2 for overweight and -2 for undernutrition). BIVA and PA investigation were performed using the bioelectrical impedance data. The BIVA classification was conducted according to the position on the ellipse of the graph related to a pre-existing reference population, which was described in the manual by Piccoli et al (7). The PA was calculated using the following formula: PA(Φ) = (Xc/R)×(180°/π).

### Statistical analysis

The sample size was calculated from an analysis of the number of hospitalizations that occurred in HCFMRP-USP in the 3 years prior to the survey (2009-2011). Different prevalence estimates and values for accuracy were considered. Accuracy is associated with the amount (percentage) considering that the prevalence might be far from real. We thus determined that a minimum sample size of 281 hospitalized children would be required for an estimated prevalence of 30% and 5% accuracy.

Descriptive statistics are presented for all study parameters (mean, median, standard deviation, minimum, and maximum). Parameters with a Gaussian or normal distribution were compared by Student’s *t* -test (comparison of age, weight, height, BMI, resistance, R, and PA). Some variables were considered logarithms to meet the assumptions of this test (e.g., weight for males aged 5 to 19 years). The software SAS 9.2 (SAS Institute Inc., USA) was used for this purpose.

The kappa coefficient was calculated to verify the agreement between the nutritional status classifications according to the WHO criteria and the BIVA software.

Correspondence analysis (correspondence maps) was applied to determine whether qualitative variables were spatially associated. This type of analysis involved a multivariate technique that explores categorical data. In this analysis, an array of non-negative data is graphically displayed as row and column points in biplots in which the vector space has a smaller dimension than the original space. In this way, we can interpret the relationships between lines and columns and between rows and columns. The geometric and algebraic mean from the correspondence map belongs to a family of available imaging techniques that are based on a lower position (i.e., singular decomposition value) in the matrix approach. In other words, the aim of this analysis was to find the subspace that best fits the set (cloud) of points in the Euclidean space. This adjustment of the subspace was performed by the weighted least-squares method, in which the generalized Euclidean distance (weighted) was used in a system of point masses.

Finally, analysis of covariance was used to eliminate confounding variables (i.e., sex, age, BMI, maternal education, type of delivery, and breastfeeding) that were previously established in the classification of nutritional status. The same software (SAS 9.2) was used for all of these tests.

### Ethical aspects

The Research Ethics Committee of the HCFMRP-USP approved this study. Signed informed consent was obtained from all participants’ parents and/or guardians. Children aged ≥7 years also provided signed assent.

## Results

The WHO criteria for BMI/A were used to classify all children according to the collected anthropometric data. The prevalence of malnutrition among hospitalized children (n=292) was 35.27%, and 16.10% and 19.17% of the subjects exhibited undernutrition and excess weight, respectively. Of all participants, 93.15% and 80.82% had their weight and height/length, respectively, measured by the ward staff (nursing technicians or nurses) according to data in the hospital system. All patients (100%) in the control group (n=234) were eutrophic according to the WHO criteria. The characteristics of the variables of interest of the two groups are shown in Table 1.

**Table:**
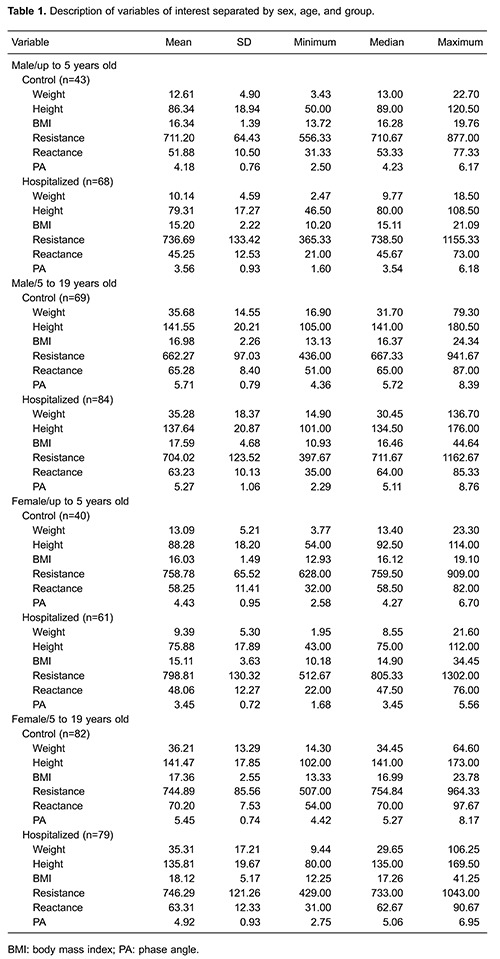

**Figure 1:**
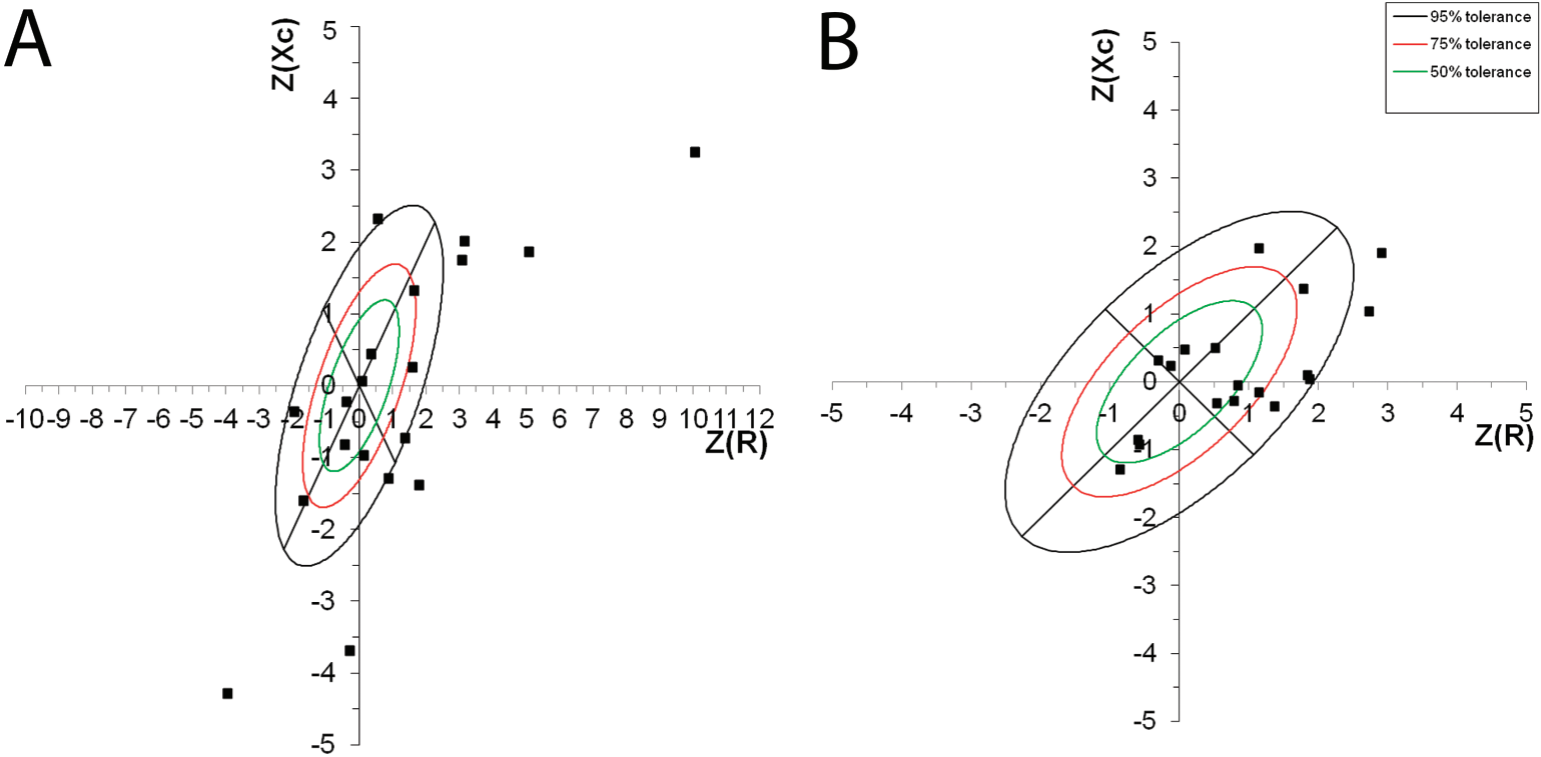
Example of the bioelectrical impedance vector analysis (BIVA) classification for female patients at 6 and 7 years of age. Classifications for the hospitalized (*A* ) and control (*B* ) groups are shown.

We were able to use the data from 447 individuals (237 pediatric ward patients and 210 controls) for the BIVA classification. It was necessary to exclude patients aged 4-23 months because no reference population existed in the program or literature at the time of analysis. In the hospitalized group, 55.45% of the patients were classified as having malnutrition (classified as thin, cachexic, or obese). More specifically, 51.25% presented with undernutrition and 4.20% had edema/obesity. Among the children in the control group, 23.34% were malnourished; 20.95% had undernutrition, and 2.39% presented with edema/obesity (Figure 1).

To correlate the BMI/A anthropometric WHO classification with the BIVA analysis, it was necessary to create three cluster categories (i.e., eutrophic, overweight, and undernourished). After the construction of clusters, we calculated weighted kappa coefficients using z-scores of -1 and -2. The methods did not show agreement because the weighted kappa coefficients for z-scores of -1 and -2 were 0.189 and 0.227, respectively.

All comparisons of PAs differed considerably. The mean differences in weight, height, and BMI were statistically different between the hospitalized and control groups in children aged 1 month to 5 years (Table 2). The PA was significantly different between the hospitalized and control groups after correcting for confounding variables (i.e., sex, age, BMI, maternal education, type of delivery, and breastfeeding) by covariance analysis (Table 3).

**Table:**
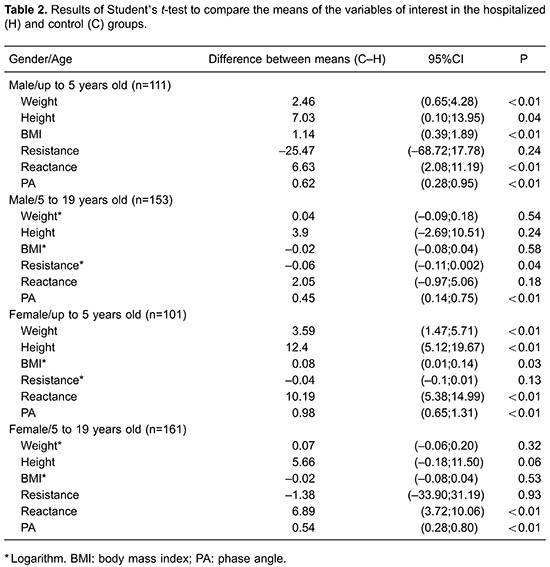

**Table:**
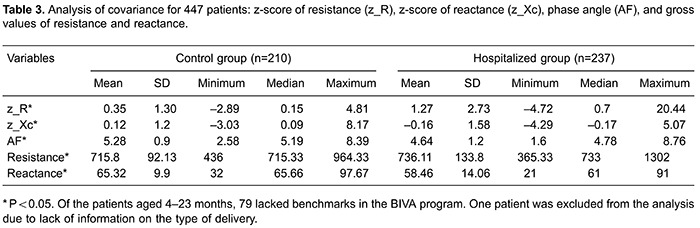

**Figure 2:**
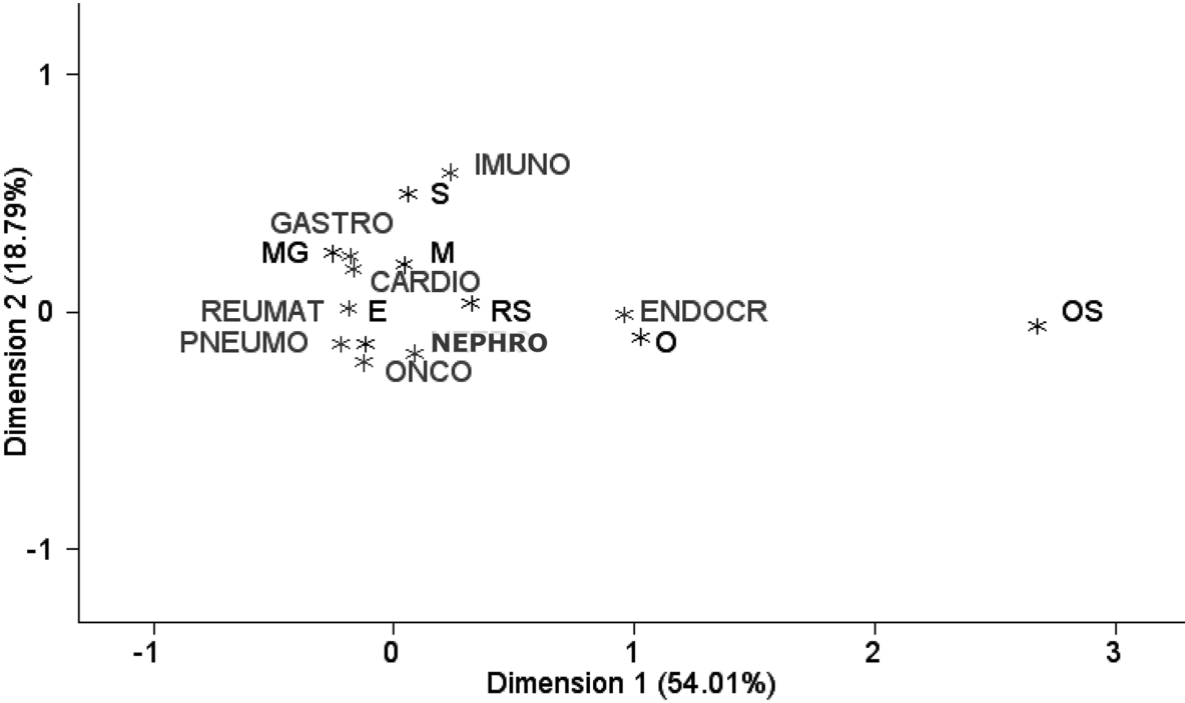
Map of correspondence for nutritional status according to body mass index for age of the patients in the hospitalized group by medical specialties. OS: severe obesity; O: obesity; S: overweight; RS: risk of being overweight; E: eutrophic; M: thinness; MG: severe thinness; IMUNO: immunology; GASTRO: gastroenterology; REUMAT: rheumatology; NEPHRO: nephrology; CARDIO: cardiology; ONCO: oncology/hematology; PNEUMO: pulmonology; ENDOCR: endocrinology.

**Figure 3:**
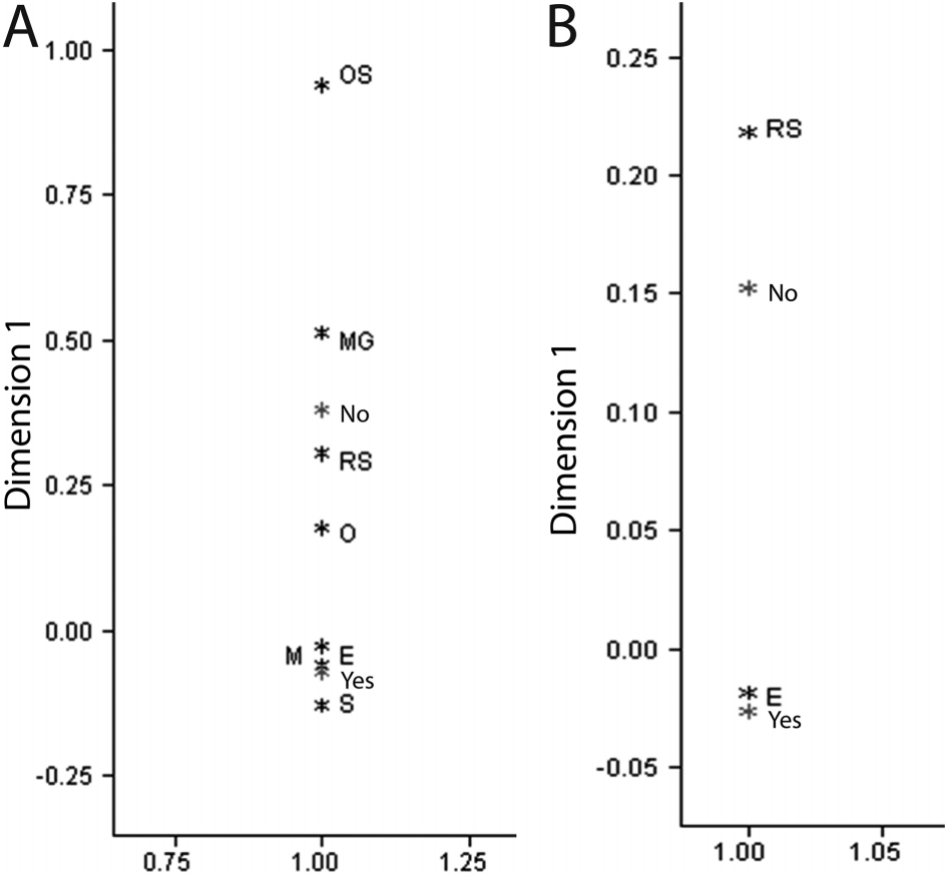
Map of correspondence for nutritional status according to body mass index for age in the hospitalized (*A* ) and control (*B* ) groups. OS: severe obesity; O: obesity; S: overweight; RS: risk of being overweight; E: eutrophic; M: thinness; MG: severe thinness; NO: not breastfed; YES: breastfed.

The correlation between the nutritional status of hospitalized patients (hospitalized group) and their nutritional status at the time of hospital admission according to their BMI is shown in Figure 2. By performing this correlation, we were able to identify the ward with the most malnourished patients. Patients who were staying in the endocrinology ward more frequently exhibited obesity. Patients admitted to the cardiology and gastroenterology wards more frequently exhibited severe thinness and slimness. Furthermore, patients were often classified as eutrophic in the rheumatology, oncology, and pulmonology wards. The correlation map indicated that breastfeeding was related to a normal weight (same point on the correspondence map). Patients who were not breastfed more often exhibited severe thinness (same point on the correspondence map) and had a risk of becoming overweight (Figure 3).

## Discussion

This is the first cross-sectional study to establish the prevalence of malnutrition in the pediatric wards of HCFMRP-USP. To analyze the patients’ nutritional status in this study, we used calculation of the PA and performance of BIVA as complementary methods. In this investigation, children in the pediatric wards of HCFMRP-USP were compared with an unpaired control group. Patients who were selected for this study had a wide age range (1 month to 18 years), which increased our sample size and made the study findings more reliable.

Analysis of the percentage of weight (93.15%) and height (80.82%) conducted by the nursing professionals in the pediatric wards allowed us to determine that data were lacking from the medical records, especially information regarding length; when it was not possible to perform both measurements, the staff did not even report estimated values. Nevertheless, the percentage of length recorded in this study was more effective than that reported in other Brazilian studies (2,6). Measuring weight and height is extremely important because these measurements can help classify a child’s nutritional status and determine the subsequent nutritional approach. Unfortunately, as mentioned in several studies, classification of the nutritional status of hospitalized children is often neglected (2). Furthermore, many authors have highlighted the need to appropriately intervene when patients experience some kind of deficit in their nutrition (12). Joosten et al. (13) stated that it is crucial to screen children at admission to prevent complications.

The prevalence of malnutrition verified in this study was similar to that of other countries. According to the literature, the prevalence of malnutrition currently ranges from 5%-27% in developed countries (3,). The prevalence of malnutrition in hospitalized children of the Ibero-American countries (16) is as follows: Colombia, 27% undernutrition and 6.3% overweight; Mexico, 12.2%; Cuba, 33.3% undernutrition and 10.9% overweight; and Argentina, 49.6% undernutrition and 1.9% overweight.

The aforementioned prevalence data have led to considerations about the nutritional transition within the HCFMRP-USP. In absolute numbers, the HCFMRP-USP has the lowest undernutrition rate among the above-mentioned studies. In contrast, excess weight is a great concern. According to data regarding the prevalence of malnutrition in a healthy Brazilian population in 2008/2009, there was an observed transition from nutritional deficit to overweight (17). The National Center for Health Statistics estimates that 16.9% of children and adolescents aged 2-19 years in developed countries are obese (18). Notably, this phenomenon can be alarming from a public health standpoint; similarly to cases of undernutrition, excess weight during childhood may have negative consequences for the prognosis of any disease and may favor the onset of chronic diseases in the future (19). Therefore, these results lead us to suggest that it is essential to provide some nutritional education (i.e., information on food types and games that increase the understanding of how and why children and adolescents should eat better) to patients at the time of hospital discharge to improve or solve the problem of malnutrition.

With respect to the use of BIA in children, Farias et al. (20) employed a standardized PA (obtained by using the PA corrected for sex and BMI for a reference population) to assess nutritional risk in 2012. These authors attained promising results and concluded that this tool was able to detect changes in the study population (patients with bone marrow transplantation) more sensitively than could the WHO criteria for BMI/A (20). We were unable to directly compare our results with those of Farias et al. (20) because the PA values were not standardized in our study. However, we can state that PA is a promising tool with which to assess the nutritional status in these transplanted children without requiring weight or height measurements. This tool also proved to be more sensitive for detecting body changes associated with undernutrition.

PA measurements in children have been used in a few published studies. However, many studies have shown that PA measurement is a good alternative method for predicting malnutrition in adults (i.e., low PA value) (21 22 23). Kyle et al. (22) examined ways to associate the PA with other malnutrition assessment methods for screening adults. These authors concluded that the PA is a useful tool for identifying nutritional risk at hospital admission (24).

The importance of using different tools to evaluate the nutritional status of children, as we have proposed, was also reinforced in a Brazilian study involving an indigenous study population (25). Guida et al. (26) demonstrated that BIVA is not comparable with BMI/A (using the WHO graphics) in children aged 8 years, especially when they are overweight. These authors highlighted that appropriate BIVA cutoff points are not yet available and stated that combining analytical tools could assist in the dietary treatment of hospitalized children (24).

In 2012, Hartman et al. (6) conducted a review of different screening tools for malnutrition and attempted to establish a correlation among the methods to unify them. However, these authors found no consensus regarding the standardization of methods, indicating that attention to nutritional status is easily overlooked.

There have been many discussions regarding the application of screening methods tailored for each specialty. BIVA is an inexpensive and current tool that is widely used at our hospital. Likewise, PA measurements can be easily calculated using the above-mentioned formula.

According to the correlation maps, the various pediatric wards should adopt distinct approaches for managing malnutrition. For example, patients in the endocrinology ward should receive nutritional education, whereas the staff in the cardiology ward should be concerned with undernutrition. This strategy could make the pediatric wards more effective. As observed in the correlation map, breastfeeding is beneficial for children with any disease. Nevertheless, the underlying diseases should not be ignored during the assessment of nutritional status in these patients.

In the present study, the authors were able to develop and propose a protocol that requires validation and subsequent implementation. The proposed screening method can improve the service in pediatric wards and aid the performance of other studies that will consistently standardize and verify whether the prevalence of malnutrition in hospitalized children is different among wards. The screening protocol has been tailored to HCFMRP-USP, and it combines the WHO methods with BIVA/PA to screen hospitalized children at admission (Supplementary Material).

The prevalence of malnutrition at the HCFMRP/USP, Brazil, is consistent with our hypothesis; however, it is possible to improve these data using other tools (i.e., BIVA and PA) despite disagreeing with anthropometry. According to our study findings, weight, height, and bioimpedance can be evaluated at admission in all children. These measures should enable early intervention in terms of diet therapy and thereby reduce the length of hospital stay. BIVA and PA should also be used in series to easily evaluate any changes in their values. Similar studies must be conducted in the future to establish new perspectives and improve the pediatric wards.

## Supplementary Material


