# Supplementary material for: Prevalence of child malnutrition at a university hospital using the World Health Organization criteria and bioelectrical impedance data
Source: Braz J Med Biol Res. 2016 Feb 2;49(3):e5012. doi: 10.1590/1414-431X20155012 (PMC4763819; doi:10.1590/1414-431X20155012)
Supplement: Supplementary file 1 [file 1414-431X-bjmbr-1414-431X20155012-S1.pdf]

Screening protocol for hospitalized children

				Points		
Name:				Zero (0)	ONE (1)	TWO (2)
Hospital record:		Date:				
Date of birth:		Age:<br>(less than 5 years old = 2 points)				
Diagnosis:						
Is it possible to measure weight? (if yes = zero ; if no = 3 points)						
Is it possible to measure height? (if yes = zero ; if no = 3 points)						
Weight (kg):		Height (cm):				
BMI (kg/m^2):						
WHO criteria classification (BMI/A)						
A) z-score less than -3; percentile less than 0.1 = 2 points						
B) z-score between -2 and -3; percentile between 0.1 and 3 = 1 point						
C) z-score greater than +3 = 2 points						
D) z-score between +2 e +3 = 1 point						
E) other = zero						
Resistance (R)			R MEAN			
1	2	3				
Reactance (Xc)			Xc MEAN			
1	2	3				
Classification BIVA						
Eutrophy and Athletic: zero						
Lean, Cachexic or Obese: 1 point						
Phase Angle [(Xc/R)*(180/3.14)]						
Less than 5 years old		PA < 4.1 = 2 points				
Between 5 and 19 years old		PA < 5.4 = 2 points				
Other values = zero						
			**SUM**			
			**TOTAL**			

Classification of nutritional status according to the combination of criteria

Zero to 2 points: minor nutritional risk, nutritional support and bioelectrical impedance on the first and last day of hospital stay

3 to 5 points: moderate nutritional risk, nutritional support and bioelectrical impedance every 2 days

6 to 10 points: severe nutritional risk, nutritional support, confirming food ingestion on main meals and bioelectrical impedance every 2 days
